# p38 MAPK in MDS

**DOI:** 10.18632/aging.100757

**Published:** 2015-06-02

**Authors:** Irene Gañán‐Gómez, Zachary S Bohannan, Guillermo Garcia‐Manero

**Affiliations:** Department of Leukemia, The University of Texas MD Anderson Cancer Center, Houston, TX, USA.

Myelodysplastic syndromes (MDS) are a group of heterogeneous hematopoietic malignancies occurring mainly in the elderly and characterized by the defective production of mature blood cells. MDS subtypes can be classified as "low-risk" and "high-risk" syndromes on the basis of their risk of progression to acute leukemia. Although progression to leukemia is the most common cause of death in high-risk patients, infections, bleeding, and other events related to cytopenias are the most common cause of death in low-risk disease [1]. Most newly diagnosed patients have low-risk MDS; however, this subtype has fewer available treatment options, and its management is focused on improving quality of life and reducing the impact of cytopenias. Alternative treatments, such as hypomethylating agents or lenalidomide, are only used for high-risk MDS or when cytopenias worsen, but even these are non-curative options. This gap in MDS therapy is caused by the lack of molecular targets owing to the heterogeneity and/or low frequency of known genetic and cytogenetic abnormalities in this group of diseases.

In this landscape, the recent accumulating evidence of the inflammatory nature of MDS has shed some light on the pathogenic mechanisms of this disease and opened the door to a whole new spectrum of therapeutic agents [2]. One of these agents, the p38 MAPK and Tie2 oral inhibitor ARRY-614, has been recently tested in low-risk and intermediate-1 MDS in a Phase I study and was well tolerated and induced tri-lineage hematologic responses in over 30% of the patients [3].

The use of a p38 MAPK inhibitor in low-risk MDS is strongly supported by the finding that these patients have increased p38 MAPK phosphorylation in bone marrow (BM) hematopoietic progenitor cells of all myeloid lineages and that these phosphorylation levels are positively correlated with the rate of intramedullary apoptosis, which is a characteristic feature of low-risk MDS [4]. These facts point to a relevant role for this kinase in the defective hematopoiesis observed in low-risk MDS. Similarly, it has been demonstrated that the pharmacologic or RNA interference-mediated inhibition of p38 MAPK significantly decreases apoptosis in MDS bone marrow mononuclear cells (BMMNC) and CD34+ progenitor cells while it significantly enhances erythroid and myeloid colony formation [4]. In line with these observations, ARRY-614 decreased the presence of phosphorylated p38 MAPK in BM and reduced BM apoptosis in most MDS patients while efficiently decreasing the levels of some inflammatory factors and erythropoietin in patients' plasma [3].

One remaining question is how p38 MAPK inhibition improves hematopoiesis in MDS. On one hand, increased levels of hematopoietic suppressor cytokines such as interferons, TGF-β and TNF-α in the peripheral blood (PB) and BM of MDS patients are well documented [2], and recent evidence that innate immune signaling in normal hematopoietic stem and progenitor cells (HSPC) indirectly regulates myeloid differentiation through the induction of cytokine-based autocrine and paracrine loops suggests that the signaling pathways triggered by those cytokines directly control the outcomes of hematopoiesis [5]. The inhibitory effects of these cytokines in normal hematopoiesis seem to rely on the activation of p38 MAPK [4, 6], so it can be easily hypothesized that p38 MAPK is responsible for the control of the gene expression programs activated by HSPC-produced cytokines and, therefore, that its deregulation in MDS exerts a direct effect on hematopoiesis. Additionally, p38 MAPK activity was shown to mediate paracrine-induced TNFα secretion in both normal and MDS BMMNC [7], which suggests the participation of this kinase in autocrine signaling loops. Because the presence of TNF-α in MDS plasma and BM is positively associated with intramedullary apoptosis rates [2], it could be inferred that p38 MAPK also contributes to the MDS phenotype by indirectly inducing the depletion of HSPC via TNF-induced apoptosis. This hypothesis is supported by the fact that the restoration of normal mature blood cells in PB upon p38 inhibition is accompanied by a decrease in BM apoptosis.

Therefore, the results obtained from the Phase I study of ARRY-614 [3] are greatly relevant for two reasons. First, the restoration of hematopoiesis observed in this study confirms the causative role of inflammatory signaling in the hematopoietic differentiation defects that characterize low-risk MDS. Second, these results show the great potential of therapeutic strategies that target common innate immune regulators rather than just one cytokine. Whereas blocking the activity of one, or even more, inflammatory factors may not have a significant biological impact owing to biological redundancy, inhibiting a signaling hub that connects several cytokine-activated pathways (e.g., inflammation, apoptosis, and differentiation), such as p38 MAPK, has more therapeutic promise for the treatment of low-risk MDS. This has added importance because of the traditionally limited treatment options that are available to this subset of patients and is an encouraging step forward on the path toward a targeted management of MDS.
